# Visual recognition of mirror, video-recorded, and still images in rats

**DOI:** 10.1371/journal.pone.0194215

**Published:** 2018-03-13

**Authors:** Tomiko Yakura, Hiroki Yokota, Yusuke Ohmichi, Mika Ohmichi, Takashi Nakano, Munekazu Naito

**Affiliations:** Department of Anatomy, Aichi Medical University, Aichi, Japan; University of Lethbridge, CANADA

## Abstract

Several recent studies have claimed that rodents have good visual recognition abilities. However, the extent to which rats can recognize other rats and distinguish between males and females using visual information alone remains unclear. In the present study, we investigated the ability of rats to visually recognize mirror, video-recorded, and still images and to discriminate between images of males and females. Rats were tested in a place preference apparatus with a mirror, a video-recorded image of a rat, or a still image of a rat at one end. The data were assessed using t-test with Bonferroni correction. Male and female rats spent significantly more time in the mirror chamber and the video-recorded image chamber than in their respective blank chambers (P < 0.05), and male rats also spent more time in the chamber containing a still image. Furthermore, it was found that male rats exhibited significantly more sniffing behavior around the mirror than in the blank chamber (P < 0.05), whereas female rats were no significant differences in the sniffing behaviors in the mirror, moving or still image experiments. Identical results were obtained regardless of whether the rat in the image was the same or opposite sex. These results indicate that rats can process the differences in mirror, video-recorded, and still images as visual information, but are unable to use this information to distinguish between the sexes.

## Introduction

It has been shown that rodents are generally non-visual animals because they form their recognition using information that they obtain through olfaction. However, it is known that rats use vision in play fighting and other close quarter social interactions [1]. Recently, some studies have claimed that rodents use visual information for social recognition [2], leading to an increased interest in visual recognition in rodents [3,4]. For example, it was demonstrated that rats can discriminate emotional expressions of pain from neutral expressions in other individuals using only visual information [5]. Watanabe et al. [6] have reported that mice that were shown videos of three conspecific social behaviors (sniffing, copulation, and fighting) exhibited a visual preference for certain social behaviors. Mice preferred the copulation video to the sniffing video, the fighting video to the sniffing video, and the fighting video to the copulation video. Similarly, other studies have shown that mice can discriminate between paintings by different artists [7] and use visual information for social recognition [2]. However, the extent to which rats can recognize other rats using visual information alone remains unclear.

In general, some studies have claimed that rats judge sex through the detection of pheromones by olfactory system [8]. A recent study has shown that there are sex-based differences in the social modulation of learning in rats [9]. Therefore, it is also interesting to consider to what extent rats can distinguish between males and females using visual information alone.

Several studies have investigated the extent of visual recognition in animals through the use of mirrors [10,11]. With regard to experiments on visual recognition using mirrors in rodents, rats showed anxiety-like behavior when they were placed in a box with four mirrored walls [12,13]. However, these studies did not include an investigation on the visual recognition of mirrors. Another possibility to consider is that the visual information may be more effective in the context of additional social cues. For example, in a test enclosure that contains rat odors, the test subject may be more motivated to ‘look’ for subtle differences in the visually transmitted information present in a conspecific. The mirror images may reflect the greater interest generated by real-time movement by the rat in the image, adding more cues to the context about the possible identity of the partner, such as olfactory cues, may elicit greater usage of the visual information.

In this study, we investigated the ability of rats to visually recognize mirror, video-recorded, and still images and to discriminate between images of males and females.

## Materials and methods

### Animals and housing

Sprague–Dawley rats (8-weeks old; n = 162) were purchased from SLC (Shizuoka, Japan) and maintained at 22°C–24°C and 50%–60% relative humidity at a 12h light–dark cycle in the Laboratory Animal Center of Tokyo Medical University for 2 weeks before use. Throughout the acclimation and experimental period, pairs of rats were housed in polycarbonate cages (26 cm wide × 43 cm deep × 20 cm high), where they had free access to tap water. The handling and care of the rats conformed to the National Institutes of Health (NIH) guidelines for animal research, and all experimental protocols involving animals were approved by the National Research Institute for Child Health and Development Animal Care and Use Committee (Permit Number: 2017–18). All experiments involving animals were performed according to these guidelines and experimental protocols. All efforts were made to minimize animal suffering.

### Apparatus

**Fig 1:**
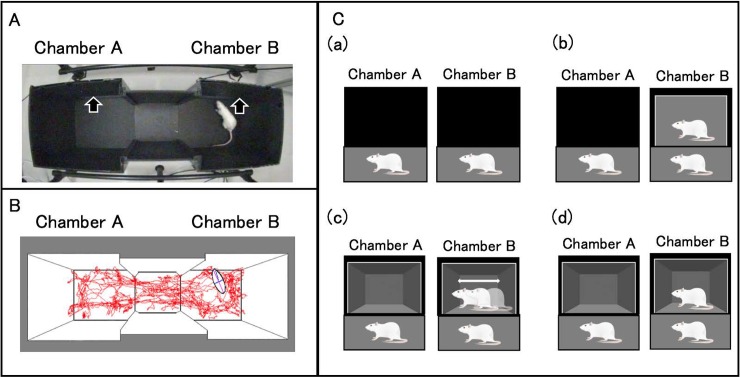
Experimental apparatus of a place preference. A place preference apparatus with a mirror, a video-recorded image of a rat, or a still image of a rat at one end. (A) Photograph of the apparatus showing the central compartment and two side chambers. (B) Tracings of the actual route one individual travelled over a 10 min period. (C) Diagram of the setup for each of the preference tests: (a) normal, (b) mirror, (c) video-recorded image, and (d) still image. Arrows indicate the position of black panel, a mirror, a video-recorded image of a rat, or a still image.

Rat was examined in a place preference apparatus using a mirror, a video-recorded image of a rat, or a still image of a rat at one end (Fig 1A). The apparatus, which has a central compartment (20 cm × 20 cm × 40 cm) and two identical side chambers (30 cm × 30 cm × 40 cm) is commonly used for conducting conditioned place preference tests [5,14]. The behavioral field was set on a black sheet to improve the image contrast of the animals against the background. Either a black panel, a mirror, a video-recorded image of a rat, or a still image was installed along the walls of the chamber (Fig 1C). The size of the black panel, the mirror, and the tablet (KPD108R, KEIAN; screen resolution, 1024 × 600) was 7 cm × 8 cm. The apparatus was placed in a sound attenuating room with a light level of approximately 100 ± 3 lux with a ceiling lamp above each compartment. A 1/3 Color CCD camera (PX70KSD; PRIMETECH ENGINEERING CORP., Tokyo, Japan) was mounted 30 cm above the center of the apparatus. The recordings made by the digital CCD camera mounted above the apparatus were analyzed using the Top Scan system software (ver. 3.00; Clever Sys., Inc., VA, USA), which detects rat movements and behaviors based on the video tracking of multiple individual body parts, postures, and the frequency of movements (Fig 1B) [15,16].

### Experimental design

The rats were placed into the apparatus and allowed to acclimate to the environment for 30 min before the testing commenced. To avoid issues with habituation, each rat was evaluated once. The floor and walls of each compartment were cleaned with 70% ethanol after each trial, and the chamber where the rat was placed was randomized. A control and three experiments (Experiments 1–3), were performed separately, as described below.

#### Control

Two black panels were placed at both walls of the chamber (Fig 1C). Thirteen male and 13 female rats were evaluated, and the position of the black panels was altered in each trial.

#### Experiment 1 (Ex. 1): Validation study of a mirror

A mirror was placed at the wall of one chamber, and a black panel was placed at the wall of the second chamber as a blank (Fig 1C). Thirteen male and 13 female rats were evaluated, and the position of the mirror and black panel was altered in each trial.

#### Experiment 2 (Ex. 2): Validation study of a video-recorded image

Two tablets were placed at two walls of the chamber. One tablet showed a video-recorded image of a rat, and the other tablet showed a video-recorded image without a rat as a blank (Fig 1C). Video images with rat were recorded by a digital video camera (HC-V360M, Panasonic) in advance. All rats were 8–9-weeks old at the time of recording and were unfamiliar to the subject rat. It is possible that the rats preferred the particular video images. Therefore, six different video images were recorded by using 3 male and female rats when the rats were doing the same social behavior as at the time when a rat was not showing a specific emotion [1,2,3]. The video-recorded images without rat were recorded under the same condition. The images were edited on a computer using Movie Maker and Xmedia softwares and transferred onto the tablet using software. These recordings had duration of 7 min and were played repeatedly on the tablets without sound (S1 Video).

To determine whether rats of the same sex can identify each other, 13 male and 13 female rats were placed separately in chambers with video-recorded image of rats of the same sex (Ex. 2A). To examine whether rats can discriminate between sexes using visual information alone, 13 male and 13 female rats were separately placed in chambers with video-recorded image of rats of the opposite sex (Ex. 2B). The position of the two tablets was traded every time, and three movies were randomized in each trial.

#### Experiment 3 (Ex. 3): Validation study of a still image

Two tablets were placed at both walls of the chamber. One tablet showed the still image of rat, and the other tablet showed the still image without rat as a blank (Fig 1C). The still images were obtained from the same recordings as used in Experiment 2 using Movie Maker and Adobe Photoshop Elements. All still images were presented without sound.

Thirteen male and 13 female rats were evaluated using still image of rats of the same sex (Ex. 3A). To examine whether rats can discriminate between males and females using visual information alone, 13 male and 13 female rats were evaluated using still image of rats of the opposite sex (Ex. 3B). The position of two tablets was replaced and three still images were randomized in each trial.

The behavior of each test rat was video-recorded for 2 h using the digital CCD camera and locomotive behavior was then analyzed offline using the software program Top Scan (ver. 3.00; Clever Sys., Inc., VA, USA). The recordings made by the digital CCD camera mounted above the apparatus were analyzed using the Top Scan system software, which detects rat movements and behaviors based on the video tracking of multiple individual body parts, postures, and the frequency of movements [14,15] (Fig 1B). The following variables were recorded: 1) the cumulative time spent in each chamber; 2) the number of sniffs to each wall; 3) the cumulative time spent sniffing each wall; and 4) the number of entries into each chamber (scored when the rat had all four feet in the chamber).

### Data analysis

Data were expressed as the mean±SEM. For all the statistical analyses t-tests with Bonferroni correction were used to assess significance between two groups. Prism software was used to analyze all data (Graph Pad Software, Inc., La Jolla, CA, USA).

## Result

### Control

**Fig 2:**
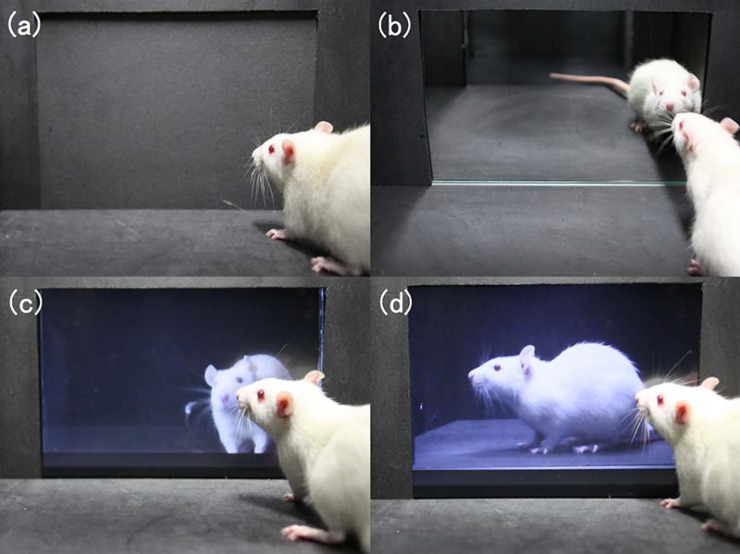
Experiments of the preference tests. Photographs of rats in each of the preference tests: (a) normal, (b) mirror, (c) video-recorded image, and (d) still image.

**Fig 3:**
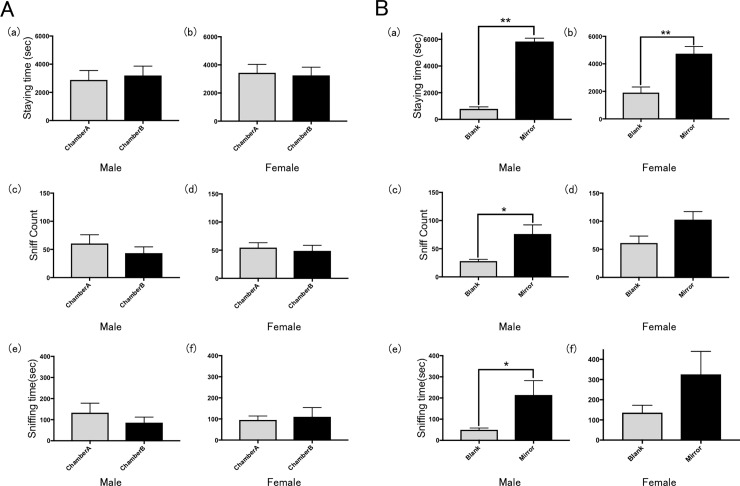
Results of control and Ex. 1. Preference of rats for (A) one of the two blank side chambers under normal conditions (Control) and (B) a chamber containing a mirror (Ex. 1) in terms of the mean time spent in the chamber by (a) males and (b) females, the mean number of times the wall was sniffed by (c) males and (d) females; and the mean time spent sniffing (e) males and (f) females. Error bars represent the SEM. *p < 0.05, **p < 0.01, t-test with Bonferroni correction.

Both male and female rats (♂, n = 13; ♀, n = 13) spent approximately equal time in the two side chambers of the apparatus (Figs 2 and 3A). In addition, they sniffed the blank screens for a similar number of times (Fig 3A). Both male and female rats spent approximately equal time sniffing the two blank screens (Fig 3A).

#### Ex. 1: Validation study of a mirror

Both male and female rats spent significantly more time in the chamber with mirrors than in the chamber with the blank screen (Figs 2 and 3B). Male rats also sniffed the mirror more frequently than the blank screen and spent significantly more time sniffing in the chamber with the mirror than in the blank screen (Fig 3B). Female rats spent more time sniffing in the chamber with the mirror than in the blank screen, but there was no significant difference in sniffing time (Fig 3B).

#### Ex. 2: Validation study of a video-recorded image

**Fig 4:**
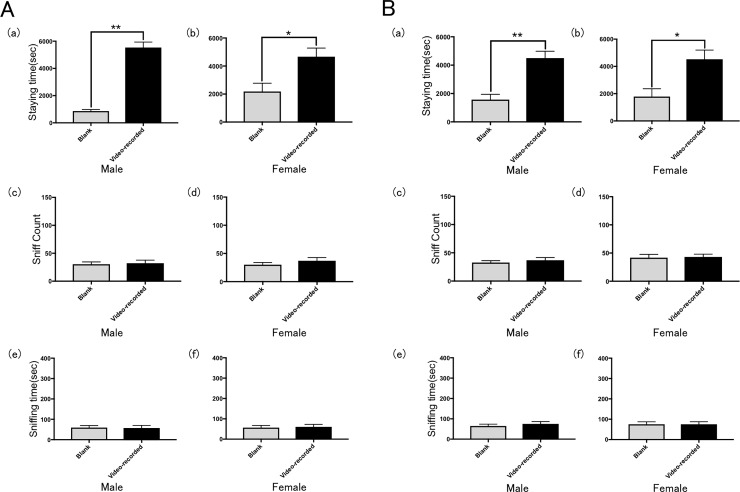
Results of Ex. 2. Preference of rats for a chamber containing a video-recorded image of a rats (A) the same sex (Ex. 2A) and (B) the opposite sex (Ex. 2B) in terms of the mean time spent in the chamber by (a) males and (b) females, the mean number of times the wall was sniffed by (c) males and (d) females; and the mean time spent to sniff by (e) males and (f) females. Error bars represent the SEM. *p < 0.05, **p < 0.01, t-test with Bonferroni correction.

When shown recordings of rats of the same sex, both male and female rats spent significantly more time in the chamber with the video-recorded image of rats than in the blank chamber (Figs 2 and 4A). However, there was no significant difference in the sniff counts between male and female rats in these trials (Fig 4A). Both male and female rats spent approximately equal time sniffing the video-recorded image and blank screens (Fig 4A).

When shown a recording of a rat of the opposite sex, both male and female rats also spent more time in the chamber with the video-recorded image of rats than in the blank chamber (Fig 4B). Again, there was no significant difference in the sniff counts between male and female rats (Fig 4B). Both male and female rats spent approximately equal time sniffs the video-recorded image and blank screens (Fig 4B).

#### Ex. 3: Validation study of a still image

**Fig 5:**
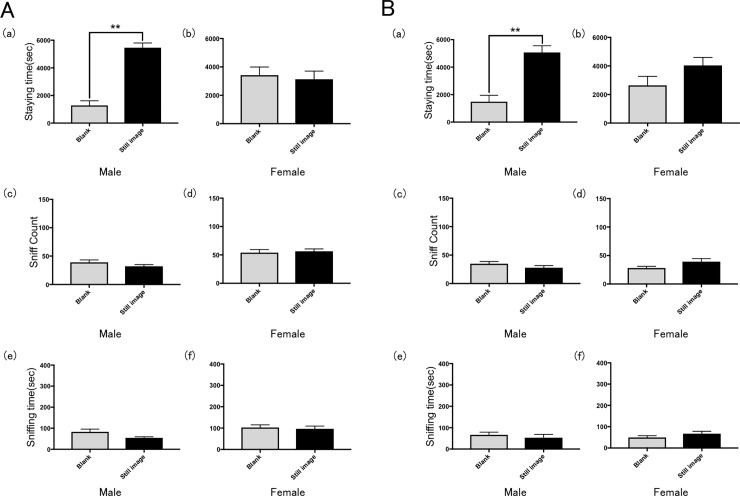
Results of Ex. 3. Preference of rats for a chamber containing a still image of a rat of (A) the same sex (Ex. 3A)and (B) the opposite sex (Ex. 3B) in terms of the mean time spent in the chamber by (a) males and (b) females, the mean number of times the wall was sniffed by (c) males and (d) females and the mean time spent to sniff by (e) males and (f) females. Error bars represent the SEM. **p < 0.01, t-test with Bonferroni correction.

Male rats spent significantly more time in the chamber with the still image of male rats than in the blank chamber (Figs 2 and 5A). By contrast, there was no significant difference in the time female rats spent in the two chambers when shown a still image of a female rat, and there was no significant difference in sniff count between either sex (Fig 5A). Both male and female rats spent approximately equal time sniffing the still image and blank screens (Fig 5A).

Similar results were obtained when a still image of the opposite sex was used. Male rats spent significantly more time in the chamber with the still image of female rats than in the blank chamber (Fig 5B). By contrast, there was no significant difference in the time female rats spent in the two chambers when shown a still image of a male rat, and there was no significant difference in sniff count between either sex (Fig 5B). Both male and female rats spent approximately equal time sniffing the still image and blank screens. (Fig 5B).

## Discussion

In this study, a mirror or tablet was used as the stimulus-presenting device in each experiment and was placed at the wall of a side chamber. The average speed for every 10 min for rats in the control group was calculated for 120 min, and it significantly decreased at 100 min (S1 Fig). Therefore, we investigated rat behavior for 120 min. Rats can process differences among mirrors, video-recorded images, and still images as visual information; however, they are unable to use this information to distinguish between the sexes. To the best of our knowledge, this is the first study to investigate the visual recognition ability of rats using mirrors, video-recorded images, and still images.

Several previous studies have explored visual recognition in rodents [5,17,18]. For example, Davide et al. [18] have obtained behavioral evidence that rats are capable of identifying objects under different viewing conditions. In addition, Nakashima et al. [4] have reported that rats using only visual information can discriminate between emotional expressions of pain in other individuals and neutral expressions. These previous experiments have used Long–Evans rats, which have black eyes, because they have better visual recognition ability than other rat strains, which have red eyes [19]. In this study, we used Sprague–Dawley (SD) rats, which have red eyes, and we revealed their ability to visually recognize differences among mirrors, video-recorded images, and still images. The ability to recognize oneself in a mirror is considered an indicator of self-recognition. It is known that humans can recognize themselves in a mirror by 18–24 months of age [20], and other animals, such as elephants and pigs, can recognize themselves in mirrors [11,21]. However, surprisingly, recent studies have also suggested the possibility that mantas and ants can recognize themselves in mirrors [10,22].

Sherwin [23] has argued that since the mirror is a vision-based stimulus, it lacks species-specific biological relevance to mice and is therefore neutral or occasionally aversive. In our experiments, when a mirror was placed at the wall of a side chamber, the rats approached and sniffed the mirror surface repeatedly (Fig 2B). This suggests a strong interest in the mirror. Furthermore, after the rats remained in front of a mirror for 60–90 min, they crouched down and did not move any further toward the front of the mirror. These behaviors did not occur when the rats were exposed to video-recorded images and still images. Therefore, it appears that SD rats can recognize mirror images perhaps because the long-time exposure to the mirror image resulted in a mental stimulus.

In most exploratory behavior models of anxiety in rats, females present with lower levels of anxiety than males [24–26]. For example, females consistently display lower levels of anxiety in an elevated plus maze; they more fully explore the open and exposed arms of the plus maze [25], and they consistently display lower levels of anxiety in open field tests [26]. We observed similar tendencies in our study with mirrors and video-recorded images (Figs 3B and 4A). By contrast, males spent significantly more time in the chamber that contained a still image than in the blank chamber (Fig 5A). This was revealed further when the females demonstrated interest in moving images (mirror and video) but showed no interest in still images. In addition, even though the images of males and females were exchanged, the almost same behaviors were observed in both Ex. 2 and 3 (Figs 4B and 5B). From these results, it appears that rats do not depend on visual information alone to distinguish between males and females.

Although this study quantitatively demonstrated for the first time that there are sex-based differences in the visual recognition behavior of rats. Some limitations must be addressed. In this study, we performed experiments from morning until noon, which is the inactive period for rats. If the experiments were performed during the active period at night using infrared cameras, the results might differ. Second, fluctuations of sex hormones associated with the estrous cycle were not taken into consideration, which might explain why it was not possible for the rats to distinguish between males and females using visual information alone. Third, it has not been verified whether rats can recognize self-mirror images in detail. In this study, the rats’ behavior seeing the mirror, video-recorded images, and still images were verified for the first time; however, if they were to see the images multiple times, it is possible for rats to learn from these situations. Further studies focusing on mirror image recognition and learning ability using rats are necessary.

## Supporting information

S1 Fig The average speed for every 10 minutes in rats of the control group. Significantly different from the average speed at 10 min, *p < 0.0001.
(PNG)

S1 Video A video-recorded image of male rat. (MP4)
