# Supplementary figures and images for: Visual recognition of mirror, video-recorded, and still images in rats
Source: PLoS One. 2018 Mar 13;13(3):e0194215. doi: 10.1371/journal.pone.0194215 (PMC5849344; doi:10.1371/journal.pone.0194215)

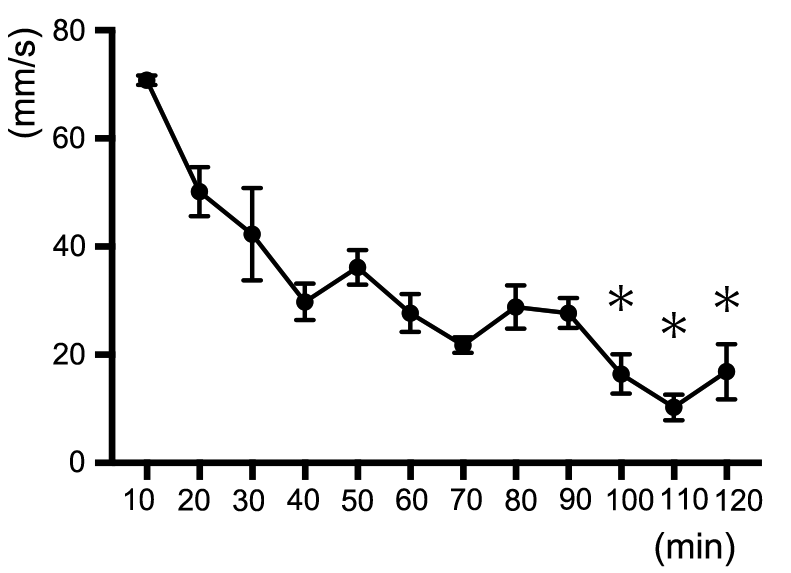

Supplement: S1 Fig — Significantly different from the average speed at 10 min, *p < 0.0001. (PNG) [file pone.0194215.s001.png]
